# Increased Noradrenaline as an Additional Cerebrospinal Fluid Biomarker in PSP-Like Parkinsonism

**DOI:** 10.3389/fnagi.2020.00126

**Published:** 2020-06-16

**Authors:** Rocco Cerroni, Claudio Liguori, Alessandro Stefani, Matteo Conti, Elena Garasto, Mariangela Pierantozzi, Nicola B. Mercuri, Sergio Bernardini, Giorgio Fucci, Renato Massoud

**Affiliations:** ^1^Parkinson Center, Department of System Medicine, University Tor Vergata, Rome, Italy; ^2^UOC Neurology, Department of System Medicine, University Tor Vergata, Rome, Italy; ^3^Department of Experimental Medicine and Surgery, Faculty of Medicine and Surgery, University Tor Vergata, Rome, Italy

**Keywords:** parkinsonism, Tau-protein, noradrenaline, CSF, prognosis

## Abstract

Academic centers utilize sequential clinical and neuroimaging assessments, including morphometric ratios, to obtain an unequivocal diagnosis of the non-synucleinopathic forms of Parkinsonism, such as progressive supranuclear palsy (PSP), however, a 1–2 year follow-up is required. The on-going long-lasting trials using anti-tau antibodies for PSP patients might therefore be biased by the incorrect enrollment of Parkinson’s disease (PD) patients manifesting early axial signs. This perspective study aimed at achieving two major goals: first, to summarize the established biomarker candidates found in cerebrospinal fluid (CSF) in probable PSP patients, including low p-tau and altered neurofilaments. Second, we share our recent data, from CSF samples of well-selected PSP subjects, attributable to both main variants (and revisited in light of MDS criteria), who were followed for 1 year before and 2 years after lumbar puncture. We found a significantly high level of noradrenaline (NE) in these patients, similar to controls, when compared to PD patients. In contrast, CSF samples, in PD, showed a significant reduction in CSF NE and its major metabolite, which confirmed that PD is a multi-system disease involving several endogenous pathways. The NE axis impairments were prominent in PSP featuring worse NPI. It might represent a counterpart to the early and peculiar psycho-pathological profiles that are observed in *tauopathies*. In conclusion, we highlight that CSF biomarkers, which are easy to collect, can provide rapid insights as diagnostic tools. Early alterations in endogenous NE machinery in atypical Parkinsonism may represent a specific risk trait in forms characterized by a worse prognosis.

## Introduction

Progressive supranuclear palsy (PSP) is a four-repeat tauopathy, whose clinical phenotype is quite heterogeneous; therefore, effective biomarkers are required to characterize the disease. Despite the fact that pathological mechanisms, in experimental models of PSP, appears well delineated (including transmission of intracellular altered tau seed from cell to cell, up to a diffuse brain deposition, Gibbons et al., 2019), our capability to transfer these models into translational or pharmacological research for PSP remains elusive. Many imaging studies in humans have reported possible PSP biomarkers, including brainstem atrophy (plus MRI index, Quattrone et al., 2018), brain pathological changes in both diffusion-weighted and tensor MRI, a reduction in cerebral [^18^F] fluorodeoxyglucose uptake, striatal dopamine imaging abnormalities, and neurofibrillary tangles of tau pathology on PET (Passamonti et al., 2017). However, a recent review by Whitwell et al. (2017), on behalf of the MDS-PSP study group, highlighted that shortfalls remain in the field because few studies could represent “*definitive* PSP biomarkers.”

In this context, collecting cerebrospinal fluid (CSF) biochemical markers may be crucial to achieving a more accurate diagnosis of PSP.

For decades, the scientific community has recognized total tau (t-tau), phosphorylated tau (p-tau), and Aβ42 (Aβ42) as biomarkers for Alzheimer’s disease (AD) diagnosis (Blennow et al., 2010; Balasa et al., 2014). CSF concentrations of t-tau and p-tau reflect axonal degeneration, neurofibrillary tangle deposition, and neuronal injury. In contrast, the concentration of CSF Aβ42 reflects senile plaque deposition and synaptic loss. Besides AD, these biomarkers (and their ratio, such as Aβ42/p-tau or p-tau/t-tau) support the diagnostic accuracy for patients afflicted by different neurodegenerative disorders. Alterations in CSF Aβ42 levels, for example, may increase the risk of developing dementia in patients with Parkinson’s disease (PD; Zhang et al., 2013; Blennow et al., 2016; Kovacs et al., 2017).

In contrast, for tauopathies such as PSP or corticobasal degeneration (CBD), primarily caused by neuronal injury driven by different isoforms of tau proteins due to abnormal phosphorylation at different amino acid groups, the cascade of mechanisms that induce neuronal loss remains unclear and the search for consistent CSF biomarkers continues.

In this article, we discuss recent findings that have shed new light on diagnostic markers for tauopathies.

## Summary of Current CSF Markers for PSP and PD

In PSP, neurodegeneration affects various brain regions, including the cortex and subcortical structures, such as the basal ganglia, cerebellum, and brainstem. Pathological changes are the result of extensive synaptic loss and dysregulation in several neurotransmitter systems, principally the dopaminergic and cholinergic networks (Williams and Lees, 2009; Gilman et al., 2010).

Interestingly, low CSF concentrations of t- and, more consistently, p-tau, have been reported in PSP patients, despite the fact that tau pathology plays a significant role in PSP pathogenesis (Magdalinou et al., 2014; Jabbari et al., 2017; Table 1).

**Table 1:** Main available biomarkers for PSP.

	**CSF biomarkers in PSP (positive or investigated)**	
p-tau	Low levels correlate with a greater disease severity and faster rate of disease progression (in RS)	Rojas et al. (2018)
	Similar reduction in PSP in iNHP	Schirinzi et al. (2015)
t-tau	Panel of nine biomarkers useful for PD vs. APD; not significant role of t-tau in PSP diagnosis,	Magdalinou et al. (2014); Magdalinou et al. (2015)
	Predict and track disease progression	Jabbari et al. (2017)
	Immunoprecipitated N-224 tau fragment lower in PSP than AD	Cicognola et al. (2019)
	**Other markers of neurodegeneration**
	Prognostic value (increase mortality)	Constantinescu et al. (2019)
NFL	Not significant correlation with t-tau	Bridel et al. (2019)
VILIP-1	Supportive of AD and progressive MCI, non tauopathies	Mavroudis et al. (2020) and Molinuevo et al. (2018)
	**Synaptic dysfunction**	
NEUROGRANIN	Reduce in “Parkinsonian syndrome” vs. AD	Hall et al. (2020)
	**Markers of amyloidogenesis and brain amyloidosis:**
sAPP-α	Lower, in PSP/CBS, with respect to AD, PD and even control	Tang et al. (2020)
sAPP-β	Suited for diagnosing early MCI/AD	Magdalinou et al. (2014)
	**Inflamation**
SAA	SAA increased in PDD and MSA, not in PSP; YKL-40 lower in PD than control;	Hall et al. (2018)
YKL-40	YKL-40 increased in PSP	Magdalinou et al. (2015) and Boxer et al. (2017)
	**Plasma biomarker in PSP**	
NFL	Increased levels of NFL from seminal reports, i.e., in ALS and FTD.	Lu et al. (2015) Steinacker et al. (2017)
	Increased diagnostic accuracy for Atypical PD vs. PD	Hansson et al. (2017)
	**Miscellanea**	
ENTERIC/MICROBIOTE	GFAP over-expressed in the enteric glial cells (EGCs) of PD patients (not AP)	Clairembault et al. (2014)
ASTROCYTES/PATHOLOGY	Increased YKL-40 immunoreactivity of astrocytes in tauopathies including PSP but also AD	Querol-Vilaseca et al. (2017)

*List of abbreviations: PSP, Progressive supranuclear palsy; RS, Richardson syndrome; iNPH, normotensive hydrocephalus; PD, Parkinson’s disease; APD, atypical Parkinsonism; AD, Alzheimer’s disease; NFL, Neurofilament light; MCI, Mild Cognitive Impairment; CBS, Corticobasal syndrome; MSA, Multisystem atrophy; ALS, Amyotrophic Lateral Syndrome; FTD, fronto-temporal Dementia; GFAP, Glial Fibrillary Acidic Protein*.

Few conditions, if any, mimic this reduction in CSF p-tau. One study has reported lower CSF t- and p-tau in patients with idiopathic normal pressure hydrocephalus (iNPH) than in controls; further, that p-tau did not differ between iNPH and PSP (Schirinzi et al., 2015).

In contrast, t-tau levels, but not p-tau levels, are modestly lower in *de novo* PD patients (Parnetti et al., 2019). However, CSF t-tau, in PD, rapidly increases with disease progression. We have shown that CSF t-tau and the CSF/serum albumin ratio, which is a valid estimation of blood-brain barrier (BBB) integrity, gradually increases in PD patients, and correlates with Hoehn & Yahr staging. Of note, these PD patients were chosen amongst non-cognitively impaired patients (Liguori et al., 2017).

Altogether, these data indicate that the significant reduction in p-tau found in PSP warrants attention; therefore, future studies should further define its clinical significance, its prevalence in specific PSP phenotypes, and its variability in PSP progression.

In addition, clinical studies have shown the potential utility of dosing plasma and CSF concentration of the neurofilament light chain (Nfl) to provide specific and prognostic insights in different diseases [consider Lu et al. (2015) for sporadic motor neuron disease and Steinacker et al. (2017), investigating Nfl levels for monitoring progressive aphasia (PPA) variants; Table 1]. In PSP, Rojas et al. (2018) showed that higher CSF Nfl and lower p-tau concentrations mark disease severity and “accelerated disease progression” (this applies to PSP Richardson syndrome—RS—variant; Table 1). No strong clinical correlation was found, relating Nfl to CSF t-tau levels (Rojas et al., 2018; Bridel et al., 2019).

It is worth considering that CSF Nfl has been associated with increased mortality in both brain neurodegenerative disorders that feature alpha-synuclein dysregulation [PD and multiple system atrophy (MSA)], and syndromes characterized by abundant tau protein deposition (such as PSP; Constantinescu et al., 2019).

In AD studies, axonal degeneration and neurofibrillary tangle pathology are reflected in increased CSF t-tau and p-tau levels (Blennow et al., 2010), in clear contrast with PSP (Wagshal et al., 2015). These divergent results may depend on the different biochemical conformations of tau proteins in the two diseases, possibly leading to a differential affinity for monoclonal antibodies that are currently used in ELISA analyses (Wagshal et al., 2015). Furthermore, PSP is defined as a four-repeat tauopathy (4R), characterized by the deposition of the tau isoform with four repetitions of the Microtubule Binding Domain (MTBD; 4R), preferentially retained within the neurons. Conversely, AD, senile dementia, as well as normal aging, show homogeneous amounts of the 4R and 3R isoforms (Irwin, 2016).

Furthermore, several groups are also investigating whether other classes of CSF biomarkers underlie PSP pathology. At first, NeuroGranin, involved in synaptogenesis, resulted in increased idiopathic PD vs. AD (hence, not contributing, so far, to ameliorating differential diagnosis amongst Parkinsonisms; Hall et al., 2020). Interestingly, sAPPα/sAPPβ, as markers of altered “amyloidogenesis,” were found to be lower in both PSP/ CBD patients when compared to both PD and AD patients (Tang et al., 2020). Finally, Hall et al. (2020) investigated the role of SAA and YKL-40, showing their increase in PD plus dementia and MSA (2018) but not in PSP. Table 1 reports a synthesis including some hints from extra-neural studies.

Our group has shown that CSF alterations in catecholamines may represent an additional diagnostic tool in idiopathic PD. We found that the homovanillic acid (HVA)/dopamine ratio was higher in advanced PD patients manifesting dyskinesia than in *de novo* PD. This suggested that there is a profound late change in DA turnover as the disease progresses (Lunardi et al., 2009). More recently, we documented that PD patients with early motor fluctuations also showed abnormal CSF HVA levels, which correlated with the wearing-OFF phenomenon (Stefani et al., 2017).

Goldstein et al. (2015) have focused on impaired vesicular storage mechanisms for decades. They stress the failure of the presynaptic machinery in PD dopamine terminals and the risk of endogenous over-production of toxic aldehydes. Interestingly, patients with PSP also showed a reduced production of DOPAC (Goldstein et al., 2015; Stefani et al., 2016), indicating that tauopathies and synucleinopathies may share disturbed presynaptic dopamine terminals. However, preliminary data indicated that the noradrenaline (NE)/Dihydroxyphenylglycine (DHPG) ratio might also be altered, shedding light on the NE pathway; therefore, this effect requires further elucidation, as documented here.

## CSF Noradrenaline in Patients With PSP and PD: Our Center Study

### Methodological Notes

Here, we report the preliminary data from our center. We recruited 15 patients with PSP, who were followed-up with, at our site, for at least 3 years (including 2 years after CSF sample collection). The Ethical Committee of the University of Tor Vergata approved this study, including formal consent released by each patient (plus qualified caregiver), including all procedures (imaging and CSF sampling).

The diagnosis of PSP was made by clinical findings and corroborated by imaging in most cases (see Table 1). Specifically, patients defined as probable PSP showed symmetric parkinsonism, no evidence of significant asymmetry on beta-CIT striatal SPECT, and a poor response to LD.

We applied the new diagnostic criteria released by the MDS-study group (Höglinger et al., 2017; Grimm et al., 2019). These criteria identify four functional domains (ocular dysfunction, postural instability, akinesia, and cognitive dysfunction) as clinical predictors of PSP. All patients in our series matched these criteria. In terms of phenotypical sub-classification:

- eight out of 15 patients were classified as probable PSP-RS (six and two manifested O1-P1-A1-C1 and 01-P1-A1-C2, respectively); two are deceased, to date.
- five patients were classified as PSP-Parkinson (PSP-P) with a dominant parkinsonian syndrome showing some LD response (<25% after 1 year, classified as A3) or a negligible response at 6 months (A2).
- two patients manifested with a mixed clinical syndrome with features reminiscent of CBD (early levitation and asymmetric apraxia); however, it was associated with repeated falls in the last 18 months (criteria P1).

In addition, the majority of patients with PSP showed midbrain atrophy (nine out of 15 were also morphometrically validated with prolonged follow-up ≤2 year after CSF collection; Table 2).

**Table 2:** Demographic and clinical features of patients with progressive supranuclear palsy (PSP), Parkinson’s Disease (PD), and control.

	*PSP* (*n* = 15)	*PD* (*n* = 14)	*CON* (*n* = 16)
Gender (M/F)	8/7	8/6	9/7
Age (years) PSP-RS/PSP-P	67.9 ± 7.6/65.8 ±4.8	67.2 ± 6.2	62.1 ± 11.5
Disease phenotype	RS = 8;	AR	NA
	PSP-P = 5;		
	Others = 2		
Disease duration (months) at time of CSF sampling	15.3 ± 8.4	21.3 ± 9.4	NA
UPDRS-III (global/27-30)	NA	28 ± 8.2/5 ± 2	NA
LEDD (mg)	175 ± 50	625 ± 275	NA
IMAGING	MRI (15/15, 9 also morphometric)	DAT-scan (14/14)	NA
	DAT-scan (11/15)		
MMSE	23.3 ± 2.8	26.8 ± 2.1	29.2 ± 0.8

*List of abbreviations: AR, Akinetic/Rigid; NA, Not applicable; UPDRS, Unified Parkinson’s Disease Rating scale; LEDD, Levodopa Equivalent Daily Dose; MMSE, Mini-Mental State Examination*.

For a control cohort, we recruited 16 patients with peripheral neuropathy and with no signs of any neurodegenerative disease as the control group (but performing lumbar puncture, for diagnostic purpose, under the same setting, see later). In previous articles, we have documented that patients afflicted by peripheral neuropathies (so-called OND, other neurological disease) in fact represent a reliable control group, given their CSF catecholamine content (Olivola et al., 2014; Stefani et al., 2015).

PD patients (*n* = 14; Table 2) matched the following inclusion criteria; diagnosis was based on UK Brain Bank criteria, supplemented by beta-CIT striatal SPECT scanning, and excellent motor responses to LD. Patients were in Hoehn & Yahr stages 1–2.5. Moreover, patients with PD were described as being attributed to the akinetic-rigid phenotype with some degree of early axial signs. Table 2 provides the main clinico-epidemiological features of the three cohorts.

All patients underwent a CSF examination. After obtaining informed consent from the patients, lumbar puncture was performed to obtain CSF samples for research purposes. Samples were assayed from 14 and 15 patients with PD and PSP, respectively, and 16 controls.

The lumbar puncture was performed at a hospital ward after 2 days of hospitalization, between 8 and 9 AM after overnight fasting. Dopamine receptor agonists were stopped at least 2 days beforehand. The patients’ therapies were modified as follows: monoamine oxidase inhibitors were discontinued at least 3 weeks in advance; entacapone, when present, and any dopamine receptor agonist (including extended released forms) were discontinued at least 2 days before; levodopa (LD)/carbidopa or LD/benserazide were held overnight.

The first 4 ml of CSF were used for routine laboratory analyses. The next 2 ml was obtained for research. The CSF was aliquoted into 2 × 1,000 μl aliquots in plastic sample tubes. We added 10 μl of 0.01 M perchloric acid to each 1,000 μl aliquot. The samples were frozen at −80°C until further analyses were performed within 2 weeks of collection.

We determined the concentration of catecholamines and derivatives in CSF using HPLC with electrochemical detection, as described previously (Holmes et al., 1994), with slight modifications. Briefly, the concentration of the internal standard (DHBA) was 50 ng/ml and a volume of 0.04:0.2 M phosphoric acid:acetic acid (20:80 v/v) was used to elute catecholamines from the alumina in 200 μl. The electrical potentials applied to the first, second, and third electrodes were 0.4 V, 0.1 V, and −0.35 V, respectively.

## Results

We analyzed L-Dopa (DOPA), norepinephrine (NE), dopamine, and 3,4-DHPG CSF levels in the three groups, PD, PSP, and control (CON; Table 3A). Comparisons between groups were performed using the Mann–Whitney *U*-test for non-parametric numerical variables.

**Table 3A:** NE and DHPG levels (nM/L) in PSP, PD and Control (Mean ± SD).

	PSP (Mean ± SD)	PD (Mean ± SD)	CON (Mean ± SD)	PSP vs. PD (*p*-value)	CON vs. PSP (*p*-value)	CON vs. PD (*p*-value)
**NE** (*nM/L*)	6.26 ± 3.19	2.15 ± 1.2	8.61 ± 4.51	0.04	ns	0.002
**DHPG** (*nM/L*)	10.69 ± 3.66	10.32 ± 3.67	11.29 ± 4.95	ns	ns	ns

*Mean differences between the groups were assessed by factorial analyses of variance carried out on log-transformed data, with Fisher’s Least Significant Difference *post hoc* test. Log transformation was performed because the standard deviation of the neurochemical measures varied withthe means. Comparisons between groups were performed using the Mann–Whitney U-test for non-parametric numerical variables*.

CSF NE levels were significantly higher in the PSP group when compared with the PD group (*P* = 0.04). In addition, CSF NE levels were significantly higher in CON when compared with PD (*P* = 0.002; Table 3A). In contrast, no statistically significant differences were found in the NE metabolite, DHPG, between the PD group and healthy controls as well as between the PSP group and CON. DA levels were not significantly different in conditions of brief washout (see methods, data not shown).

Further, we explored the Neuropsychiatric Inventory Questionnaire (NPI) scores to measure an ample panel of behavioral symptoms in PSP patients (Cummings, 1997). The score for each dimension ranged from 0 to 12, with a maximum total score of 144 in the 12-item version (Binetti et al., 1998, for Italian validation). We dichotomized the patients with PSP as Neuropsychiatric symptoms (NPS)-positive (PSP/NPS+) and NPS-negative (PSP/NPS−) based on the NPI cut off score ≥4, as previously used in clinical trials and observational studies (Birks and Harvey, 2018). We compared CSF data between PSP/NPS+ and PSP/NPS−.

Table 3B shows that there was a significant increase of NE levels in NPS+ patients.

**Table 3B:** NE levels in PSP patients, distinguished in two subgroups based upon NPI scores (cut-off score ≥4).

	**NE** (*nM/L*) (Mean ± SD)	*p*-value
**PSP/NPI+** (*n* = 9)	12.29 ± 4.59	0.001
**PSP/NPI−** (*n* = 6)	3.2 ± 0.64	ns

## Discussion

PSP represents a severe neurodegenerative disease that is in need of pharmacological therapies. Anecdotal experience with stereotactic neurosurgery, by delivering low-frequency deep brain stimulation of the pontine tegmentus in a PSP patient, indicated safe procedures but inconclusive results (Brusa et al., 2009; see also Hazrati et al., 2012). Promising trials with anti-tau antibodies were either halted (as the ABBV-8E12 protocol) or are awaiting disclosure.

The PSP galaxy includes different clinic phenotypes, such as PSP-RS, PSP-Parkinsonism (PSP-P), and PSP with pure akinesia, whose definition requires a multi-disciplinary approach. Several PSP patients manifested signs reminiscent of concomitant synucleinopathy. Similar to idiopathic PD, for instance, PSP patients may have an altered cortical plasticity, as shown by Conte et al. (2012) utilizing intermittent and continuous theta-burst stimulation. Moreover, similarly to synucleinopathies, Parkinsonisms due to tauopathy may show a moderate deficit of vesicular storage in dopamine neurons (Goldstein et al., 2015). Dopaminergic therapy is frequently prescribed in patients with PSP-P manifesting asymmetric hypokinetic signs, especially in the early phases. However, clinical follow-up clarifies the poor and transient LD response. Therefore, the opportunity to address any ambiguities in achieving the early diagnosis of PSP is of paramount importance.

In 2015, Magdalinou and co-authors proposed a panel of nine biomarkers to discriminate atypical PD from PD with dementia. In recent years, different reports have suggested more specific biomarkers to improve PSP diagnosis. The significance of lower CSF p-tau and higher CSF and plasma Nfl levels in patients with PSP, when compared with controls was documented. Aside from p-tau and Nfl concentrations, other studies are investigating to what extent biomarkers implied in different cascades involved in neuroinflammation and synaptic efficiency may also play relevant roles in PSP pathogenesis.

Furthermore, as we have presented in this study, patients with PSP exhibit higher concentrations of CSF NE when compared with patients with PD.

Hence, a limited, but already enriched panel of biomarkers is currently available, at relatively low cost, to improve the accuracy of early differential diagnosis between PD and PSP, when clinical presentation is not unequivocally distinguishable.

With regard to PD, we have previously shown the stage-dependent increase of CSF t-tau levels (Liguori et al., 2017), the alteration in DOPAC and HVA concentrations (Lunardi et al., 2009; Stefani et al., 2016, 2017). Here, our data demonstrate a low CSF NE content in PD. These data may reflect the concurrent involvement of multiple neurotransmitter systems in PD pathology (Eldrup et al., 1995; Chiaravalloti et al., 2013).

Regarding PSP, our preliminary data on CSF catecholamines may add to the present literature for PSP diagnosis. Although we included a small cohort of PSP patients, their diagnosis was checked by clinical follow-up, comprehensive neuropsychological evaluation, and neuroimaging.

The higher NE CSF levels detected in our PSP patients, when compared to PD, might explain the higher occurrence of NPS in patients with PSP. Accordingly, recent literature has emphasized a peculiar early neuropsychiatric profile in PSP patients, including apathy, anxiety, and perturbation of face recognition (Pontieri et al., 2012; Pellicano et al., 2017; Assogna et al., 2019). In keeping with these data, we showed a positive correlation between CSF NE levels and NPI scores. Consistently, PSP patients complaining about more pronounced behavioral disturbances may present higher CSF NE concentrations and become eligible for specific pharmacological interventions for psychopathologies.

We are aware of some limitations in this study which suggests the requirement for further investigation.

First, DHPG CSF levels did not differ between PSP and PD patients, at differences with the increase of CSF NE. A previous study reported that clozapine treatment in schizophrenic patients increased plasma NE levels (Breier et al., 1994). In that study, the authors argued that plasma DHPG levels were not simultaneously “increased because of increased NE spillover rather than decreased uptake, metabolism, or clearance” (Elman et al., 1999). Nonetheless, an increase in the NE/DHPG ratio in our PSP patients suggests that an NE increase reflects a compensatory mechanism.

Recent PET studies utilizing ^18^F-AV-1451 (Passamonti et al., 2017; Nicastro et al., 2020) described a widespread increase pattern of tau, not only in basal ganglia and dentate nucleus of the cerebellum, but, notably, also in the midbrain. Of note, immunohistochemistry studies, in post mortem tissues, have documented the degeneration of locus coeruleus in PSP (Kaalund et al., 2020), which was not easy to reconcile with our findings. Yet, although such a LC loss of pigmented neurons correlated with disease severity, the degree of neuronal loss did not correlate with tau-positive inclusions (Kaalund et al., 2020), almost as if two processes are obeying to different time-scales (being secondary the LC degeneration).

Finally, this cross-sectional study cannot permit the understanding of the progression of NE impairment in PSP patients. Extensive investigations, based on longitudinal CSF sampling collection, will provide a more conclusive interpretation.

## Conclusions

This brief opinion article has sought to summarize the currently available literature on biofluid markers supporting the diagnosis of PSP vs. PD with prominent axial signs. Furthermore, the concept that focuses on “unaltered” NE in PSP was also introduced. It is our belief that a combination of biochemical analysis and careful clinical evaluation may avoid the improper delay in PSP diagnosis and eventually ease pioneering trials.

## Ethics Statement

The studies involving human participants were reviewed and approved by the Ethical Committee of the University of Tor Vergata. The patients/participants provided their written informed consent to participate in the study.

## Author Contributions

RC: conception and design of the study and in charge of PSP follow-up. CL: writing and in charge of PSP follow-up. AS: responsible for the project and in-patients and data analysis. MC: blind execution of statistical analysis and CSF sample collection. EG: neuropsychological (and behavioral) rater. MP and SB: writing and editing. GF: competence in CSF data analysis (HPLS) and CSF sample collection. RM: responsible for biochemistry lab and data analysis. NM: Provided strong support in the development of the study and in the revision, facilitating the final manuscript.

## Conflict of Interest

The authors declare that the research was conducted in the absence of any commercial or financial relationships that could be construed as a potential conflict of interest.
